# Baron Rolfe's Charge in the Case of the Boy Allnutt—The Plea of Insanity in Criminal Cases

**Published:** 1848-04-01

**Authors:** 


### THE JOURNAL

OF

### PSYCHOLOGICAL MEDICINE

AND

### MENTAL PATHOLOGY.

APRIL 1, 1848.

### Analytical Reviews.

ART. I.—*Baron Rolfe's Charge to the Jury, in the case of the Boy Allnutt, who was tried at the Central Criminal Court, for the Murder of his Grandfather, on the* 15*th December*, 1847.

So long as it continues to be the practice of our law to refer to the same jury the very different issues of sanity and commission of crime, it is of the utmost importance to instruct the class from which our common juries are taken, in the meaning and proper application of the test of sanity prescribed by the judges—that at the time the accused commits the act, he must be conscious of the difference between right and wrong. The press has teemed with publications on the subject, some of which we have formerly noticed. The forensic subtlety of our lawyers, and the metaphysical dexterity of our modern philosophers, have combined their aid to complicate the discussion, and leave the uninitiated in hopeless perplexity.

That it is a subject of great difficulty is sufficiently apparent from the fact that the collective wisdom of our judges, who, taken as a class, may justly be considered the most learned and sagacious of the community, has, after solemn debate, been unable to provide any other test of sanity than one, which, in the popular sense of the words, partakes of almost childish simplicity, and, in their ethical sense, involves the most mysterious faculty of the human mind.

"Before a plea of insanity," say the judges, "should be allowed, undoubted evidence ought to be adduced that the accused was of diseased mind, and that at the time he committed the act he was not conscious of right and wrong."

The unconsciousness of right and wrong is, we presume, intended to be understood as an essential element in that disease of the mind which absolves from legal responsibility; and we are thus relieved, to some small extent, from the great difficulty of making the symptoms of a diseased mind intelligible to those who are inexperienced even in the very inferior art of collating the symptoms of bodily disease alone; but still, what is meant by this judicial dictum, "consciousness of right and wrong"?

If the expression intends no more than a recognition of the power of prohibition or command, and the obligation to obedience when such power is exercised by proper authority, no new light is given us. The capacity of a child to give evidence has long been tested in our courts by this criterion. Does he understand that in swearing to an untruth he is violating a command of God, and thereby incurring an awful responsibility?—which may be translated into the very words of the judicial dictum, "is he conscious of right and wrong?" All nursery discipline is regulated by the same principle; it is the test of naughtiness in swaddling-clothes. Such a test may be applicable to cases of natural idiocy; or to that comparatively rare form of insanity in which, by gradual steps, the perceptive power of the intellect has become so exhausted as to reduce man to a state of idiocy. But in all such cases the insanity is self-apparent, and no evidence of a diseased mind could be required beyond proof that the apparent physical infirmity is not assumed for the occasion; the vacant stare, the drivelling lips, the unmeaning ceaseless laugh, may all be assumed by an accomplished actor; but if they have been the chronic characteristics of the accused, they may be fairly taken as self-evidence of an idiotic, and therefore an irresponsible being. The opinion of the judicial bench could never be required in solemn deliberation by the highest tribunal in the country, to inform the public mind on such a point as this; we must assume that the test of a diseased mind by "consciousness of right and wrong," is proposed for cases of insanity in which neither idiocy nor imbecility is apparent—where the reasoning faculty still exists, but is exhibited in a manner so unusual as to warrant a doubt of its existence in a healthy and unimpaired state. To such cases of partial insanity the application of the prescribed test can never be satisfactory as a conclusive test, nor even materially assist the judgment of a jury, unaided by professional opinion.

Before we consider the character of the test, it may be convenient to inquire into the nature of that partial insanity which is to be decided by it.

A well known distinction has been drawn between folly and madness—that the fool argues wrongly from right premises, while the madman arrives at a right conclusion from premises manifestly erroneous. Making just allowance for the laconic antithesis, there is much truth in the observation; it is the perversion of reason, not the want of it, that denotes imperfect insanity. We discard all scientific language as inconvenient to our present purpose; we wish to give such a view of the question as may be intelligible to all readers; and on this principle we ask, what is understood by insanity in its perfect form? The question admits but of one answer—a total deprivation of the reasoning faculty. It may be a permanent deprivation, or only temporary; it may be accompanied by melancholy or by excitement; it may recur periodically or at uncertain intervals; it may arise from idiosyncrasy or casual injury; whatever may be its form or its source, perfect lunacy consists in the *total* deprivation of the reasoning faculty, so that while it exists, man is reduced from his humanity to a condition worse than that of inferior creation, for he becomes a mere animal without a conservative instinct. It is not improbable that such was the exact condition of Nebuchadnezzar—an absolute cessation of all mental power, unmitigated by instinctive guidance even in the search of food.

*Perfect*, or *total insanity*, such being its character, necessarily secludes the unhappy patient from all social intercourse, excepting only with his privileged attendants: he is placed out of the pale of society; and so excluded, because he is no longer *man*, though he still wears the human form: his affliction is only curable by miracle, and the same Almighty Power which first gave, and then adeemed his intelligence, can alone restore it; the close attention of his keeper may guard him from self injury; the reason of others, in short, may supply the more urgent necessities of his human condition, and nurse him for a return of rationality, but mental restoration is beyond the gift of human art, because the disease is attended with the total disorganization, we might almost say the extinction, of the functions of the brain itself, a loss as impossible to supply as a leg or an arm after amputation; the physician can *cure*, but he cannot *create*.

This is perfect, unqualified insanity, and from the nature of the case, it is one which rarely, if ever, comes under the eye of our criminal courts: such wretched beings are invisible to the world, and the world invisible to them: they are divested of responsibility by strict confinement, which places them beyond the power of offence; or if by chance they escape from their confinement, the responsibility is rightly thrown upon those whose vigilance they have deceived: but these extreme cases are, happily, of rare occurrence, compared with the many in which the light of reason, though shaded, is not entirely obscured.

This, then, is also a class of insanity for which the judicial test cannot be intended: there remains but a third—namely, where reason has not been deficient from birth, or totally extinguished by disease, yet shows itself in such an unusual manner as to render it doubtful whether it retains sufficient power to regulate the conduct. A homely simile will illustrate our meaning. A domestic cannot be held responsible for any damage he may cause in groping for an article in a darkened room, where light is denied him; if limited to the use of a lantern, or of a candle already half extinguished, the charge of carelessness in demolishing a cabinet of antique china will be unreasonable, when the light allowed suffices only to make darkness visible. The sufficiency of the light is the measure of responsibility; but it is a measure of very delicate graduation; scarcely perceptible, except by the daily remark of scientific eyes. Our judges have endeavoured to construct a phrenometer for the public information, as our instrument-makers adapt their barometers to vulgar comprehension, by writing "fair" and "rain" on the dial-plate. "Consciousness of right and wrong" forms the "change" of their phrenometer, and with unfeigned respect for their profound knowledge, we still hesitate not to say, that such an exponent of the character of the mental atmosphere is as arbitrary and as liable to error as the index of the mechanic.

Where reason is not wholly lost, or ab initio wanting, ratiocination, though in an imperfect form, will always be observable. It may express itself obscurely, it may be incoherent and inconclusive, it may argue not only illogically, but absurdly, but still it will be reasoning, and indicate a perceptive intelligence; whether such a perceptive intelligence consists with irresponsibility, must depend on circumstances which no single and strictly metaphysical test can determine either to exist or to be wanting.

This imperfect possession of the reasoning faculty, usually termed "partial insanity," may arise from various and even antagonistic causes. It may be the result of original malformation, though indicated by no decisive symptoms till the patient attains adolescence or maturity: it may spring alike from precocious or tardy development, either of the intellectual or the animal powers; it may result from the over-exertion of a mind naturally vigorous, or from the weak indulgence of an indolent, inert disposition: it may follow upon a course of ascetic self-denial, no less than upon profligate self-indulgence: it may be traced to hereditary taint, or to constitutional disease, or to accidental wounds: it may be caused by the extremity of sudden joy, or by the pressure of sorrow long endured with resignation. It would be easy to extend this alternating chain by almost innumerable links; but briefly to sum up the whole, it may be asserted, that such is the intimate and mysterious relation of the body to the mind, that, on the one hand, there is scarcely any conceivable injury to the former which may not by possibility derange the powers of the latter; nor any strong passion or emotion that violently agitates the latter, which does not more or less act upon the nervous system, or the organic functions of the body. We believe it now to be an admitted axiom, that all insanity whether perfect or partial, except monomania, is attended, if not occasioned, by bodily disease. We shall revert to this hereafter.

It must be borne in mind that we are referring to cases of acknowledged, not assumed insanity; where it is doubtful whether the accused is playing a part, we can only help forensic dexterity to remove the mask, and it is difficult long to maintain the artificial character with consistency. The question to which we confine ourselves at present is, the amount of responsibility that ought to attach to real, but yet partial insanity.

Unprofessional men, and even many who are educated for the profession, but not experienced in this branch of practice, are too apt to overlook a very important point, in forming their judgment of the sanity of a given subject. All insanity is progressive, except natural idiocy. Nemo repente rabiosus, is as true in mania as in morals: a faulty reason may first betray itself by inconsistency; and relatives or family connexions, always naturally averse even to suspect sanity of mind, carelessly observe that, "Papa forgets himself; his memory is not so good as it used to be." Inconsistency proceeds to absurdity, and absurdity becomes singularity. The same filial or fraternal feeling, made up partly of honest affection, and partly of selfish reluctance to contemplate the dreadful possibility, attributes all to "unwonted absence of mind," or "affected eccentricity," to anything rather than incipient lunacy; till at length some wild and violent behaviour forces the abhorrent conviction on their minds. Hence it is often, perhaps generally, the first real paroxysm of the malady which leads the sufferer to the bar of justice. Up to that moment, he has been thought "odd," his behaviour has been "strange and unaccountable," for a long time "he has not been like himself;" but no specific observations having been made, no notes taken of his acts or his sayings, and the paroxysm being over, even the medical witness feels at a loss to speak confidently to his state. It is extremely possible that the very novelty of the position in which the party accused finds himself, the true though shadowy impression that he has acted criminally, the array and bustle of the court, and the unwonted scene before his eyes, may combine to generate a degree of caution and restraint which confirms the belief that he is entirely under the control of reason, and therefore responsible; the medical witness is himself often staggered by this self-possession; obliged to answer promptly, he answers doubtfully, and hence his opinion passes for nothing.

This cursory review of the progressive character of partial insanity will greatly assist in explaining the variety of form which the malady assumes in its inchoate or imperfect state. It is a reasonable inference that the first subject on which the sufferer betrays perversion of intellect will bear a close relation to the source of that perversion. Perhaps disappointed hope is (of all moral, as opposed to physical causes) the most prolific source of mania. Disappointment assumes a breach of promise, either express or implied; the mind morbidly revolves the promise and its violation, dwells on the vexatious consequences, and the perfidy that has led to them, mingling self-reproach for having placed an overweening confidence, and for time and labour and money which, in that confidence, have been expended. Lost in vain attempts to account for such treatment, the patient suspects that he is the victim of intrigue and manœuvre; he must have been slandered by enemies, or betrayed by friends, or supplanted by rivals: yet he knows not where to fix suspicion; he is unconscious of an enemy, he has heard of no rival; and in his perplexity, distrust of friends seems the readiest solution. From such a state of feeling it is an easy transition to jealousy and hatred even of his nearest relations; and because he distrusts them, he will not explain his feelings; he regards their sympathy as hypocritical, their counsel as insidious, and their unanimity as conspiracy. This idea being once imbedded in his mind, he broods over it in solitude till he finds satisfaction in no other train of thought, and is immovable in conviction that he is right; the very effort to dissuade fortifies him in resistance, for it appears a new attempt to deceive him. In a word, he labours under an incurable delusion of his own creating: yet he is not slow in detecting the impression which he has conveyed to others, that his reason is affected; he knows that he is pronounced mad, yet he remains conscious that he is endowed with the reasoning faculty, and therefore quotes this dictum of his family as further and stringent evidence of an hostility that will stop at nothing. On other topics he will converse coherently, and perhaps rationally and well; he will discuss the political questions of the day with temper and judgment; he will analyse the causes of a commercial panic with shrewdness; he will even read the proceedings in a commission of lunacy, and weigh and fairly appreciate the evidence; but advert, however slightly and carelessly, to his own position, and he will either button himself up, or launch into angry invectives and furious anathema. If the malady is not arrested, and opportunity serves, this unnatural hatred breaks out in open violence against one of the suspected parties, and he deems the retribution just, though the next minute he will step a yard out of his way to avoid treading on a worm. This would by many be called monomania; it is more properly, partial or incipient insanity.

Let us take another case of frequent occurrence—religious mania. Some great domestic affliction befals a man whose education in spiritual matters has either been wholly neglected, or carelessly conducted. For the first time in his life, he practically feels the insufficiency of human consolation; some pious friend directs him to religion, as the only sure resource; and with a heart well prepared by the softening touch of sorrow, conscience is speedily awakened. The same pious friend, with the best intentions, but with less judgment than zeal, hastens to avail himself of the "favourable crisis," and puts into his hands works well calculated to awaken the impenitent sinner, perhaps, yet little adapted to the comfort of a mind already bowed down by grief. The novelty of such reading interests him; the subject is new and monopolizing, for the thoughts naturally flow towards eternity when death has removed the dearest objects of earthly affection; but as he reads, he finds sin painted in colours so strong, and its consequences portrayed with a pencil so dipped in horrors, that terror and despair begin to agitate him; the same ill-judged solicitude that put the volume in his hands, assures him that this is the legitimate and prescribed course of true repentance, and thus aggravates, instead of soothing the alarm. Let us not be misunderstood as in the slightest degree depreciating the doctrine, or questioning the heinous offensiveness of every sinful indulgence in the sight of God; *sed nunc, non erat his locus:* such doctrine has often unhinged a mind not naturally too strong, and for a time depressed by long anxiety and real affliction. It is the very nature of religious impressions, when sincerely felt, to absorb the mind, to the utter exclusion of all but indispensable worldly occupation; conscience having once taken the alarm, becomes morbidly sensitive; the new convert begins the work of reform and self-denial by abjuring amusement as vanity, and recreation as a snare; he turns his back at once, not only on old and questionable habits, but on old and unquestionable friends; he finds that they are "worldly men," and it his duty to "wean his affections from the world;" unconsciously to himself, he becomes taciturn and morose; he withdraws more and more from the humanizing intercourse of society, and finds himself shunned as he shuns others; he *hermitizes* in his own drawing-room, and finds a penitentiary in his study. It is too often the case that religious fervour thus ill directed and unseasonably excited, leads the understanding astray, and terminates in mania. In those stages we have been describing, it would be difficult to fix on any single isolated fact conclusive of a wandering mind; the symptoms, thus far, are more of a negative than a positive character, being found rather in the desertion of accustomed and undoubted duties than in irrational conversation or actual eccentricity of conduct. But they speedily betray themselves in more unequivocal form: hitherto the patient has maintained a kind of mysterious reserve as to his newly acquired opinions; he is conscious that they would not be understood, and deems them too sacred for familiar discussion; but as they become habitual, he seeks to force them upon others; and if they are questioned, resents such a reception of them as a personal insult: from conversation he proceeds to preaching, and often affects a miraculous conversion and a holy mission: he quotes Scripture with accuracy but not with point, and propounds doctrine with a fluent rapidity that shows it to be as unintelligible to himself as it is to others. His anxious family will now observe with pain that his nights are sleepless, though his days are all excitement; his appetite becomes irregular, and his person neglected; nor can his restless thoughts be fixed, even momentarily, on the state of his affairs, however urgent or important; even domestic affection seems to lose its hold upon him, and domestic cares are given to the winds. In this state of mind, he is undoubtedly the victim of partial insanity; reason has not fair play; it is not gone—it is not even impoverished: if you can once break the spell—a work of more than ordinary difficulty, for he is spell-bound by conscience—he will converse on any other topic with his former good sense; but touch on religion, and rationality is flown.

Let us take a third case, where the cause of insanity has been hereditary and constitutional; and it may be right to observe that neither this nor the preceding examples are invented to sustain a theory; indeed, every medical man can quote many of similar character that have occurred within his own practice; they are of too ordinary occurrence to need the authority of names. A young gentleman, of some reading and considerable talent, was domesticated in the writer's family; he had been under confinement a year previously to his visit, but the fact was very improperly concealed by his friends, and he came unsuspected. For more than a week, he proved a very agreeable and intelligent visitor; he was steady and regular in his habits, and lively in conversation. There was only one circumstance that excited notice—he was an excellent chess-player, extremely fond of the game, and equally vain of his unusual skill. He was absent one evening at the dinner party of a friend; some casual matter led to great excitement, under which he left the house; he got into an affray in the street on his return home, was taken to the police station, and the next morning showed such unequivocal signs of madness, that, by the advice of a medical man, he was at once conveyed to an asylum, even before notice was given to his friends. The paroxysm subsided, and in the course of a week, on visiting him at the asylum, we found him almost restored to his former self-possession; he conversed rationally, and comported himself with calmness; but though the extreme violence which he had at first exhibited did not return, it became necessary, before a month had elapsed, to issue a commission of lunacy against him. Previously to taking this decisive step, it was expedient to repeat our visit. He was apparently in the same state of calm self-possession, and, with the physician's permission, we played a game at chess; he played with his usual skill, and was successful; but the test was too severe for him; he immediately assumed the imperial character, this having been throughout the prevailing delusion of his mind, and he acted despotic royalty with so much truth, that he was obliged to be replaced under restraint within an hour. It appeared that the illusion had been generated by the game; two years elapsed before he was restored to his friends, but chess-playing was still interdicted.

These and similar cases are usually classed under the head of monomania, but, as we apprehend, erroneously; for it seems to be common to all insanity (*exceptis excipiendis* as where it can be dated from organic injury) to betray itself in its commencement on some isolated and peculiar subject. Monomania, it is true, intends a distortion or obliquity of reason on a single topic; and so far it corresponds with insanity in its incubation; but there is this essential distinction between the two classes, that monomania—if it must be considered as a malady at all, is not necessarily accompanied by bodily ailment, nor marked by the same progressive character as partial or imperfect insanity.

The course of genuine insanity appears to be this: it begins, as in the instances that have been quoted, with self-created delusion invented by the mind when under the impulse of strong, ungovernable passion. If the malady is unchecked by judicious treatment, the delusion becomes the cherished object of the thoughts. It is probable, indeed, that at the outset, the man himself is not entirely unconscious of absurdity, and dwells more and more on the fancy that he has conjured up, in vain but honest attempts to dispel it by trying to test its truth by reason: in the effort to do this, he perplexes and entangles himself, because, in his excited state, his reason is not equal to the task. The delusion gets firmer hold of him every day, till at last it monopolizes his thoughts, not only to the exclusion of every other topic, but till it absolutely incapacitates the mind for the reception of any other; and then his lunacy is perfect. If it is not too great presumption to attempt to fathom such a profound abyss as that of the human mind when tottering in its seat, we may venture to doubt whether, even in this state of absolute incapacity, the *faculty* of reason is really gone. It seems more probable, on grounds strictly metaphysical, that the faculty remains while life remains, but that the exercise of it is precluded by the utter impossibility of recalling attention from the all-engrossing subject, whatever it may be, that has acquired entire possession of the mind. This is, at all events, a theory that will account for many of the phenomena that perplex, not only the common observer, but the professed psychologist. The perfect lunatic will, no doubt, express himself incoherently on every subject as well as on that of his delusion; but this may be either because he has by long habit contrived to adapt every incident and every person to the fancy that haunts him; or because he cannot abstract his attention from the phantom of his imagination, so as to bestow it for a single moment elsewhere. Even the most rational people will at times commit themselves by gross absurdities where attention is wanting; an "absent man," as it is termed, would be deemed insane, and justly, were it not an easy matter to awaken him from his reveries, and recal him to the business of the hour.

But this feature is wanting in cases of monomania, properly so called:

the monomaniac is not absorbed by his delusion: he not only will converse rationally on ordinary matters, but is often only reserved on the single subject of his delusion, as if conscious of his mental infirmity, and apprehensive of self-exposure. This taciturnity, however, is not peculiar to monomania, for a reserved and silentious habit generally attends all partial insanity, where the malady is accompanied by depression, as in cases of melancholia; but an exemption from irreclaimable abstraction seems to form a broad distinction between monomania and other classes of the disease, not less remarkable than its stationary character and comparative enjoyment of bodily health.

Monomania is perhaps still more distinguishable from other members of the same family, if traced up to its probable source: an habitual self-indulgence of a weak or a criminal disposition. The term of moral insanity has often been used to indicate such cases, but the expression is vague and indefinite. Much of the obscurity in which the subject of mania appears to be involved, arises from this adoption of laconic and arbitrary phrases, which, even to medical men, do not uniformly convey the same idea. If by "moral insanity" is intended merely an obliquity of mental perception, as to the precise boundary between morality and immorality, there are very few people who can stand acquitted of some taint of moral insanity, as we shall presently show; it is, in fact, only the self-delusion of hardened conscience. But if the expression refers to moral, as opposed to physical causes of mental derangement, it may be doubted whether it is applicable to any case but that of monomania, as in all other forms of insanity moral and physical causes seem to combine.

Of all instances of monomania, this most frequent, and the least equivocal, is hypochondriasis. This is usually classed by medical men either among nervous or dyspeptic diseases; and, in its advanced and determinate form, it is properly classed with morbid affections of the body. Sometimes, too, there can be no doubt that it is caused by dyspepsia, and the mental derangement is removed, when the physical disorder is relieved; but hypochondriasis, in its commencement, and till it has existed long enough to generate bodily disease, is undoubtedly monomania, arising from the habitual self-indulgence of a weak and timorous disposition.

Parental, and especially maternal fondness, has educated the child to entertain undue anxiety for health; exposure of any kind is to be avoided as hazardous; a casual cold is treated as an insidious enemy; every accidental interruption of the animal functions excites uneasiness, and the family adviser is summoned; wet weather, night air, or the draught of an open window is eschewed as not less noxious than the miasma of Sierra Leone, while a cut finger or a fall sets the household in alarm. Such mismanaged nurseries maintain the carriage of the general practitioner, and often find an equipage for the physician, for the way is thus prepared for the access of hypochondriasis when the child attains maturity, and forms his own diagnosis. Health is the grand object of all his care; his thoughts are monopolized by its preservation; he vigilantly watches every change, feels his pulse twenty times a day, studies his tongue and his complexion in the glass, and never ventures on a walk without consulting the vane and the barometer; the druggist's counter is more valuable to him than the butcher's shop, and a heated and curtained bed-room safer than the cheerful, unclouded canopy of heaven smiling on a frosty day. This is the first stage of hypochondriasis, nor can it be denied that its direct tendency is to create substantial food for its morbid apprehension; yet such is the facility with which the body adapts its functions to injudicious habit, that not only in this, but in a more advanced stage, the bodily health remains unshaken, though the disease of the mind increases. We have known an instance of a person of the age of thirty, and in robust health, taking to his bed and the doctor, first for a day or two together, and eventually for weeks or months, with little interruption, till at last he died at sixty, not from any general decay or malignant attack, but from local disease, brought on by long confinement in a reclining posture. It was not till the last year of this bed-ridden life that sleep or appetite failed, or that reason was visibly affected except on the subject of health; and there he piteously bemoaned himself as a wretched hospital patient, while he indulged in five hearty meals a day, and grew obese upon the indulgence.

It is found with hypochondriac subjects that their delusion is not confined to any particular malady—on the contrary, they will often believe themselves to be, at one and the same time, the victims of diseases of an opposite and even antagonistic character; nor does this delusion spring from absolute ignorance of the science, or from the errors of imperfect knowledge. They can perfectly understand when symptoms are contradictory or fallacious; but their fallacy or their incompatibility is never admitted in their own case, though they will reason upon them sensibly, and draw just deductions in the case of another. Examples might be quoted where the patient exhibits a consciousness, not of the fanciful nature of his own ailments, but of their ultimate origin, and will warn others against the self-indulgence of over-nursing and over-weening anxiety for health; yet if the same reasoning is turned against himself, and an effort is made to dissuade him from yielding to imaginary alarm, he turns away with the look and language of incredulous despair. This is the second stage of confirmed hypochondriasis; and up to this stage it is insanity on a given subject, and, *ex hypothesi*, independent of bodily disease; for where disease actually exists, there can be no delusion on that point, though its nature may be mistaken by the sufferer. In the third and final stage, where a deluded imagination has worked out for itself a fatal reality, disease becomes progressive, and the disorder of the mind becomes in turn, confirmed by its own effects: the insanity, thus aided by bodily disease, assumes a definite and perfect form, and monomania is merged in total lunacy.

The access of monomania, where it arises from the habitual self-indulgence of a criminal disposition, may be as perceptibly traced as in yielding to the folly of a weak mind. It is the character of all vice to be progressive and self-cumulative: the axiom is so trite, that, from the school-boy dogmas of our copy-books to the moral lessons of the pulpit, it is constantly enforced upon our minds; yet the practical value of it in psychological discussions has been strangely overlooked.

Men are restrained from the commission of those acts which are, for convenience, designated by the term "crimes," by various considerations, all of which may be reduced to fear of God or fear of man; and for similar convenience, we may call the first religious motive, and the last moral motive. The excess of either motive, beyond the regulation of sober reason, may lead to precisely the same result as the utter absence of either. Conscience, the "governor" of religious motive, may acquire a vivacity of sensibility that leads to extravagance and absurdity, unless checked in time by the admonitions of a sound understanding; an extravagance that passes current for insanity with those who cannot comprehend the eccentricities of a conscience of morbid tenderness. A lady, long since deceased, was travelling in her own carriage, unattended by a servant; she was a woman of strong intellectual power, but on principle, nursed her conscience to an exquisite degree of sensitiveness; as she approached an inn where she was about to change horses, a beggar ran by the side of the carriage, entreating alms. She stopped the post-boy, and inquired into the man's story; she received the usual tale, that he was out of work and returning to his family, who lived some twenty miles further on the road. She gave him a shilling, and drove on. In reflecting on his story, some painful doubts occurred to her, whether she had acted in the true spirit of Christian charity. Was it right that she should have contented herself with bestowing a few pence when a father and a husband was walking, almost barefoot, to join an expecting family, and she herself was travelling in the same direction in the luxury of her own carriage, and that carriage half empty? She continued in this train of thought, mingled with much self-reproach, while changing horses; and during the time that this operation required, the beggar continued on his route and again was in advance of her: when the carriage passed him a second time, he gratefully touched his hat, and, attracting her notice, it occurred to her that there was the unexpected opportunity of satisfying the demands of conscience. She stopped the post-boy a second time, and desired him to open the carriage door, and invited the beggar to take a seat. Though astonished at the welcome, the man did not hesitate a moment; the boy closed the door, and they proceeded; but now the lady's conscience entered on a different course of agitation; she had done only what duty seemed to require, but had she consulted prudence? Her companion was very dirty, and might be infected by some contagious disorder! She eyed him closely, and shrank as far as possible into the corner of the seat. "Or he might have an infectious fever!" she at once opened the windows, to admit a free circulation of air. "Or, possibly, he might be mad!"—this hypothesis was too much for her nerves: she begged and implored him to jump out immediately; she shouted to the post-boy to stop; the poor man, quite convinced of *her* insanity, echoed her shouts from the other window, and the post-boy, equally persuaded of the insanity of both, drove on, regardless of their common entreaties, till he arrived at a house where he could summon assistance! This singular anecdote we had often heard, and the lady herself acknowledged to us its perfect accuracy: her name, if we felt at liberty to mention it, would be a sufficient voucher for the honest correctness of the confession. This is an instance of religious motive, not ruled by discretion, leading to conduct which any common observer, ignorant alike of the motive and its power, would deem insanity.

But it is a theory which does not depend on individual cases or anonymous authority; the history of all national religion abounds in instances of the extravagances of misguided religious enthusiasm. Our Saviour interceded for his murderers, because "they know not what they do;" St. Paul assented to the death of Stephen, believing that he was doing God service; our own annals are stained with the cruelties of bigoted priests and sovereigns, whom in charity we cannot but consider lunatic; nor would it be difficult in these days to mention many who would, with equal constancy, light the fires of martyrdom, or themselves be bound to the stake, if modern civilization had not annihilated such opportunities of exhibiting religious frenzy.

The unregulated exuberance of moral motive (as we have defined it) will have a similar effect: an eager thirst for distinction—that is, when coveted apart from its utility, for honour or popular applause—will lead an ardent mind into enterprise almost superhuman, and certainly not limited by rational expectation. To pass over many instances that will spontaneously occur to any man familiar with public men and public affairs, let any page of our military history be opened, and it will tell us of many who would face certain death for the chance of being enrolled in our list of heroes. Ancient history boasts of similar records not limited to military exploit; fathers have condemned their children to death, and executed the sentence with their own hands, to merit honour as the ministers of justice!

But if the misguided excess of religious or moral motive tends to consequences, which, when tested by common sense, we are compelled, *malgré* our admiration, to regard as absurd extravagance, much more obvious is the theory that the absence of either motive, reducing man, as it does, almost to bestial nature, will produce unequivocal insanity. Take the case of action uninfluenced by any motive whatever, and we have obviously that form of mania which we term idiocy; or take the case of action confessedly governed by bad motive, and we call it not mania, but simply wickedness resulting in crime. An extreme case, not altogether imaginary, may illustrate this position; assume that the ultimum supplicium of death is entirely removed from our statutes, and secondary punishment substituted for it in our convict colonies; the incorrigible convict, whose criminality is so incurable that he is handed over to Norfolk Island and a drum-head court-martial, as the only safe provision for the peace of a convict community, is confessedly beyond the reach both of moral principle and legal coercion, when capital punishment no longer exists. He laughs at minor penalties, however severe; they have all been tried, and tried in vain; such a being is self-removed beyond the pale of law; if not restrained for life by actual fetters, he may stab his associates, and murder at pleasure all to whom he can approach; mankind has repudiated him, and he feels at liberty to avenge himself on mankind. We have said that the case is not wholly imaginary; we vindicate the assertion, by referring to the well-known tale, placed on parliamentary record, of six convicts who effected their escape to the bush, having no arms but a single axe. Reduced to starvation, they sacrificed each other, to satisfy the cravings of hunger, till the number was reduced to two, and then possession of the axe became the only security for life; of course, it soon followed that one alone survived, and that miserable survivor returned to Hobart Town, if we rightly remember the scene of the tragedy, not to save existence, but to secure a milder death, by the hands of the executioner, than he could expect from hunger in the wilderness, and when too feeble and emaciated to terminate his own existence.

In such an extreme case as that which we have supposed, how can it be questioned that it would be a case, not of mania, but of utter bestial depravity? A total desperate defiance of all obligation, moral and divine, resulting from the habitual indulgence of a criminal disposition. Yet the outward indications of such a reprobate mind are precisely the same as the modern monomania usually displays.

These, however, are not the cases that perplex our courts. The perplexity arises, when criminal action is induced by motive which all men of ordinary intelligence reprobate as bad, but which the offender himself deems innocent, and perhaps laudable. This forms the defence which lawyers call monomania, and the test of consciousness of right and wrong is invented to decide the measure of legal responsibility that attaches to such cases. As we read the judicial doctrine, it amounts to this; that where a perversion of moral principle is sufficiently established by evidence, monomania exists, and irresponsibility must be conceded, but that a consciousness of right and wrong is conclusive against the alleged perversion of moral principle. We apprehend that this judicial proposition is erroneous in all its parts. A perversion of moral principle may, in our view, exist without monomania; may consist justly and properly with legal responsibility, even in some cases that are strictly monomaniacal; and we hold that the proposed test is, from its nature, wholly inapplicable to the case of true monomania. We feel such dissent from authority so imposing to be almost profane: yet we think we can vindicate it to the satisfaction of our readers.

We have quoted a case of hypochondriacal monomania, springing from an habitual indulgence of a weak and timorous disposition; let us trace in a similar way the monomania which proceeds from the habitual indulgence of a criminal disposition. An irresistible love of pilfering is not an uncommon, though not the most frequent form of genuine monomania; it has its source in a covetous disposition habitually indulged; it begins in infancy. The child covets a something, not for the immediate gratification it may afford him, as in the case of an apple or a toy, but from an inordinate desire to possess that something as *his own*, and this desire prevails the more as he sees it highly valued by its owner. He will steal a sovereign, though conscious that he cannot account for the acquisition of such a sum, or exhibit the purchases he may make with it; he therefore hides it, and gloats over it in secret. If speedily detected, severely punished, and judiciously admonished, his first offence may prove his only one; but should he escape detection, it is only the prelude to bolder attempts. After frequent impunity, even detection and punishment will not restrain him, but they will teach him greater caution, and as he attains mature age, and begins to understand the legal liabilities that he incurs, he will, without relinquishing the habit, confine his depredations to the property of those whose affection or relationship will forbid a prosecution; his father's watch, or his brother's purse, will be appropriated, or even the snuff-box or trinket of a domesticated visitor; but it will be observed that he invariably secretes his booty, neither seeking to sell it, nor to use it as his own. If charged on suspicion, he will lie and equivocate like a practised thief; if actually caught in the act, or with the property in hand, he will pretend mistake, and invent a dozen plausible excuses, yet he will not reform; and on the contrary, will extend his crime at last to shop-lifting and other larcenies wholly unaccountable consistently with his education and station in life. Instances are not rare where friends are under the wretched necessity of maintaining a surveillance over the culprit, and attending him in his daily walks to put shop-keepers on their guard, or pay them on the instant for articles that he purloins from the counter. Yet the same individual would disdain to cheat at cards, or to make away with property frankly intrusted to his care. His propensity is that of the magpie, to appropriate and secrete, and not to supply the means of extravagance or sensual indulgence. We have known the habit to exist, combined with much generous and self-denying disposition, and with a full and just perception both of moral and religious principle in other duties. It is from such a subject that we have taken our sketch.

It cannot be doubted that such a case is one of genuine monomania; there are none of the usual indications of felonious intention; there are none of the ordinary inducements to crime, and except in the earlier stages, there is none of the shame and confusion that detection invariably produces, unless in the hardened and professed criminal. It falls strictly within our definition of the genuine malady, arising from the inveterate indulgence of a criminal disposition.

Let us compare this case with one of spurious monomania of kindred origin. A man indulges habitually in a moody, resentful disposition; his temper is morose, his taste cruel and perverted; instead of checking such passions, he encourages them by attending prize-fights, public executions, and other brutal exhibitions; he becomes callous to suffering, dissatisfied with the world, and disgusted with existence. Excitement, whether morbid or legitimate, loses its power; he wanders through the swarming streets in self-created solitude, self-exiled from the common feelings of humanity, a prey to conscience and remorse for wasted time and ill-spent talents, yet morally incapable of extrication from his misery till suicide, in an evil hour, suggests relief. At first the thought appals him; he repels it with horror; but the horror is transient; misery continues, and the thought returns. Again the struggle is made, but he offers less and less resistance. "Is not his life his own? Is not his misery his own? Who will be injured by his death, or who will lament his loss?" And by such shallow reasoning, he silences the still lingering voice of conscience, and terminates existence. This, too, is a species of monomania arising from the habitual indulgence of a criminal disposition; yet who will dare to call it irresponsible, or vindicate the felonious act?

All crime may, in some sense, be said to belong to this class of spurious monomania; for what does criminality imply, but that passion has got the mastery of reason—that the importunity of temptation is too clamorous to allow the voice of reason to be heard? How constantly it happens that the dying convict warns his associates against the habits which conscience tells him have brought him to his end! Could any man at the first assault of vicious seduction, rationally and soberly weigh all consequences, the probability of detection, the infamy of exposure, the certainty and pain of punishment, our prisons would be comparatively empty. The criminal foolishly and irrationally speculates on impunity, and thinks immediate gratification well worth the risk of future penalty. What is this but the subjugation of reason to vice? He is, in fact, mad in his favourite pursuit. The doctrine may be carried further still. The enthusiast in an honest cause is often inaccessible to reason; the advocate of a benevolent theory is deaf to all argument against it; the ingenious mechanic will beggar himself on wild speculation or impracticable inventions; the imaginative artist will strive to give form to the chaotic fancies of a bewildered brain. It may be contended that there is scarcely an occupation in this world of business in which men do not, more or less, indicate an irrational mind, when followed up with ardour and honest zeal; but though they may thereby lose their reputation for good sense, reason is not inferred to have lost her seat.

Our conclusion is, that in true monomania there is no perversion of moral principle, except in the particular subject of delusion; and that even on this subject, there may be a consciousness of right and wrong, and yet such consciousness is not necessarily a true exponent of the monomaniac's state of mind; while, on the other hand, there exists a spurious form of the same malady in which the consciousness of right and wrong has been obliterated by habitual self-abandonment to vicious dispositions, and superseded by a general perversion of moral principles; but where, nevertheless, it would be absurd to relieve the offender from legal responsibility; and if we appear to indulge in too large an inference from the two or three cases that we have given as our premises, we reply that these cases are fair examples of the grand features of all monomania; and that every instance of the malady, whether genuine or spurious, and in whatever peculiarity of form it may exhibit itself, may justly be classed under the definition we have given of its origin, the habitual self-indulgence in weak or in criminal dispositions.

But there remains another view of the judicial test on which we are bound to explain our dissent; is this criterion of moral perversion such as, in any case, is fairly applicable? In cases of idiocy, or of perfect insanity, we have admitted it to be applicable, but have questioned the necessity of any test at all; and we think we have shown that in all cases of monomania, whether genuine or spurious, it amounts to no test, if taken only in its popular sense as intending a comprehension of that which is prohibited by competent authority, and of the obligation to obedience thereby imposed. There is, however, a third class of offenders who cannot be considered either lunatic, imbecile, or monomaniac, for whom the judicial test is probably intended.

It is said that there are men who are not the victims of any particular delusion, as in true monomania, nor in that progressive stage of general mania, which we have described as imperfect insanity, where a mist, at first partial and limited to a particular subject, gradually overspreads all the field of intellect, involving every thought in obscurity and confusion, who, nevertheless, are of diseased mind because their moral ideas are so perverted, that right becomes wrong, and wrong is moulded into right by such power of ratiocination as they still retain.

So extensive is the variety of form, not only of mental disease, but of mental character, even in its sound state, that it would be presumption absolutely to deny the occurrence of cases of this class; but we believe it to be a class so limited in number as to be incapable of arrangement by any distinctive symptoms which medical experience can define. Where bodily disease is simultaneously detected, corresponding in its features with the usual symptoms of progressive mania, the delusion of the mind may, in its earlier stages, be betrayed as well by this as by any other peculiarity; where reason strays, it is impossible to predicate the direction of its rambles, or to limit its wanderings, when their course is ascertained. In the absence of such bodily disease, we should require very unexceptionable evidence indeed, to convince us that such a case properly belongs to any family of mania.

It is obvious in the first place, that an aptitude for the distortion of truth, and an ingenuity in defending immoral conduct on moral principles are the traits of every corrupt mind; many, perhaps most men, first resort to sophistry to beguile themselves and silence conscience; they then try the same experiment on others to palliate vicious propensities that have grown beyond the power of concealment: the intemperate man pleads, for his excuse, anxiety and depression, and argues that wine was given to gladden the heart; the sensualist will quote the polygamy of the Jews, under a theocracy, as a divine permission for the unlimited indulgence of his passions; the thief will vindicate robbery by distress, and maintain with natural justice that starvation is paramount to law; the liar will contend that deception is the natural self-defence of the weak against the aggressions of the strong, and a legitimate resource prescribed by reason herself, for rational beings alone can avail themselves of it; the miser will extenuate his avarice as a prudent provision for the future; the spendthrift will affect the impulse of a noble generosity; and even the murderer will cloke his malignity as a just desire of retaliation for injury to himself or others.

And it is to be observed, that the offenders are usually, not insincere in these convenient sophisms; they have first thus reasoned with themselves in the same manner, and so frequently, that they believe in the doctrine which they preach; the perversion of their principles is honest, not affected; and so thorough is the conviction, that if they fail in extorting assent, they do not ascribe the failure to the fallacy of their logic, but to the dulness and perhaps to the hypocrisy of their opponent. Another illustration of perverted principle, honestly and soberly indulged, may be found in the polemics of divinity: how infinite are the varieties of creed and the gradations of doctrine! How contradictory are the practical conclusions to which they lead! How frequently opposed to the moral tendency of all sound religious faith! Yet they are all, in the honest judgment of their respective supporters, built on scriptural foundation, and well sustained by gospel truth.

We apprehend that no lawyer would venture to assert that, in any of these instances, the moral depravity of an opinion, or the honest obstinacy with which it might be maintained, or the mischievous ingenuity with which it might be defended, could be safely taken as conclusive of such a diseased perversion of moral principle, as to demand the concession of irresponsibility: could such a theory be established, at least one half of mankind would be virtually exempt from all legal obligation.

But besides this close resemblance between insanity of the kind supposed, and moral depravity common to all mankind, the alleged mania, if we may judge from those cases which our criminal courts are constantly bringing before the public eye, is not consistent with itself. There may be consistency in absurdity, as well as in good sense. As we never met with an instance of this form of mania in private life, which could not easily be solved into a case of simple depravity, we are obliged to form our diagnosis from the reported evidence; now we cannot recal to mind a single case, in which evidence appears to have been tendered that the accused was equally insensible of the fallacy of his moral perception, whether he was the actor of the injury, or himself the sufferer. When a man steals the property of another *on principle*, he will, on the same perverted principle, be indifferent to the invasion of his own; when *on principle* he cuts his infant's throat to translate him to heaven, he will, for similar reasons, be grateful to anybody, or even to the law, that will cut short his own existence; or more naturally still, he will accompany murder with suicide: if the insanity of perverted moral principle leads him to fire a neighbour's barn, it may be expected that he will have fired his own cottage at least a dozen times. It is one of the most common traits of genuine insanity, that the instinct of self-preservation is lost: in the monomania of hypochondriasis, this instinctive feeling is carried to a morbid extent; but in perfect insanity, whether in its state of maturity or incubation, insensibility to danger, and even to acute pain, is a very frequent symptom. The patient is often alive to injury, and vindictive in resenting it; but then it is injury repugnant to his deranged principles. Let his delusion be that of imperial sovereignty, and he resents harsh treatment as a wrong to his dignity; let him fancy himself a martyr, persecuted to the stake, and resentment is lost in self-complacency at suffering. This equity of perverted principle seems always wanting in the evidence on such cases.

Again, a partial perversion of moral principle appears to us a solecism in ethics, yet in all these cases the perversion is said to be restricted to the particular offence which brings the lunatic to the bar. All the dogmas of morality appear to be so intimately allied, so inseparably linked together, and mutually dependent, that it is difficult to conceive a perversity that holds murder to be an act of duty, founded on moral principle, yet reprobates intemperance or dishonesty, or incendiarism, as a violation of morality and religion. The Thug is an assassin, acting, as he conceives, by divine impulse; but the same doctrine teaches him to rob as well as murder, and to lie that he may do either with impunity; and so it is with the Irish spirit of the day. They steal arms as well as life, and do both, as it is said, under the stimulus of religious excitement; in such cases there is moral consistency in immoral principle; a consistency, we may observe, that has never led our courts in either country to charge for an acquittal on the ground of insanity; yet even this symptom of the alleged disease is never presented here. Why is robbery a crime? Because it is injurious to society, and therefore prohibited by law; because it is not only injurious to man, but results from the indulgence of unruly desire, and is therefore prohibited by God. We can conceive it barely possible that a man, otherwise healthy and sane, may, by perverted reasoning, arrive at the opposite conclusion; but we cannot conceive it possible that such perversion shall honestly exist on the *single* subject of robbery, and not betray itself equally on murder, or other heinous guilt; for this would imply that he has, and can justly apply, correct ideas of the peace of society and of the power and will of God; while the insulated perversion rests on the assumption that he misconceives both the one and the other.

That perversion of moral principle which is supposed to form mental disease though independent of defective organization or bodily ailment, if distinguishable from habitual depravity, is not traceable to any intelligible cause, and therein differs from all other acknowledged forms of insanity.

To say that the mind of man is by nature perfect, so far as consists with finite capacity and finite opportunity, is to say no more than that he is a being superior to brute creation. We can draw no other broad line of demarcation between human nature and bestial nature than this; that reason in the mere animal is restricted to a fixed routine, not allowing of the operation of a free will, and therefore irresponsible; animals have the same desires, the same passions as ourselves, and pursue the same means of gratifying them; but their instinct is chained down by prescribed limits, which it cannot transgress, though within those limits experience may improve the acuteness of the instinctive faculty. Human reason, on the other hand, is, in reference to our finite condition, perfect: man can pile deduction upon deduction, and find data for further inference at every stage of his ascent, and exercise a free will throughout. It would be difficult to suppose that a faculty so splendid should be conferred by Almighty power on any being of his creation, and yet be unassisted in its developement, or left, as Locke teaches us, to the accidents of casual perception; it would scarcely have consisted with the attributes of perfect benevolence and infinite goodness, to have released our reason from every bond except future responsibility, without deigning to guide our will by pointing out the distinction between right and wrong. Hence, before the fall, though the knowledge of good and evil was withheld as a matter of practical experience, it was intelligibly explained by the simplest of all moral propositions, the duty of obedience to command proceeding from unequivocal authority. Had Adam been left without the alternative of obedience or disobedience, or some analogous test of right or wrong, he could not, so far as his nature before the fall has been revealed, have been a responsible being, but must have been governed, like inferior creation, by a fixed routine, though gifted with higher powers. Practically and personally, he had no knowledge of good and evil; but he had a conception given to him (for unless it was bestowed, he had no means of acquiring it), that a certain act was wrong because it was forbidden, and yet that it rested with himself to do it or to refrain; this was, in other words, to invest his reason with freedom of action, subject only to responsibility to him who gave both the reason and the will.

If we are correct in this view, then consciousness of right and wrong is intuitive, and philosophy, unaided by revelation, has arrived at the same result. Cicero expresses it thus—"erat enim ratio profecta a rerum naturâ; et ad rectum faciendum impellens, et a delicto avocans; quæ non tum denique incipit lex esse, cum scripta est, sed tum cum orta est; orta autem simul est cum mente divinâ." And in another work he enters more minutely into the character of this divine impulse. "Est quidem vera lex, recta ratio, naturæ congruens, diffusa in omnes, constans, sempiterna; quæ vocet ad officium jubendo, vetando a fraude deterreat, quæ tamen neque probos frustra jubet, aut vetat; nec improbos jubendo aut vetando, movet; huic legi nec obrogari fas est, neque derogari ex hac aliquid, licet; neque tota abrogari potest; nec vero aut per senatum aut per populum, solvi hac lege, possumus. Neque est quærendus explanator aut interpres ejus alius, nec erit alia lex Romæ, alia Athenis, alia nunc, alia posthac; sed et omnes gentes, et omni tempore, una lex et sempiterna et immortalis continebit; unusque erit communis, quasi magister et imperator omnium, Deus ille, legis hujus inventor, disceptator, lator."

Without diving more deeply into metaphysical research, we are warranted in assuming that a distinguishing perception between right and wrong is an innate idea given with the faculty of reason itself, and which seems to form the boundary line between reason and instinct; the very perfection of instinct, as contrasted with the infirmity of reason, strengthens our position, because it implies that the first is still under the immediate control of unerring power, while the last is regulated by the infirmity of human knowledge.

It naturally follows from these premises, that the supposed malady of perverted moral principle cannot be dated from nativity, or traced to malformation from birth, unless the faculty of reason itself has been at the same time withheld; but this would not be a perversion of moral principle, but an actual and self-exposing deficiency of intellect, falling within the definition of idiocy.

What other source, then, can be found for the lunacy of the class alleged? *Ex hypothesi* it is unattended by disease, for it is the want of all evidence of bodily disease that renders some other test essential: we are alike forbidden to classify it with habitual depravity, for that is not a perversion of, but an insensibility to moral principle, being the very case that calls for legal punishment,—nor can it spring from the self-indulgence of a timorous disposition, for it is, from its very nature, a hardy defiance of all conventional morality. We can trace such a malady to no intelligible origin, and are therefore compelled, on this ground also, to be very sceptical as to its existence, though the science of psychology is not yet sufficiently advanced to deny the possibility of such a case.

However this may be, we feel (and we repeat that it is with unfeigned reverence for the dignity and the learning of the judicial bench that we express the feeling,) that while the test, in its popular sense, is unnecessary where it is most applicable, in its ethical sense, where alone it seems necessary, it is inapplicable. We will suppose the case actually to occur where the faculty of ratiocination remains, but no bodily disease being apparent, the test must be applied. To apply it efficiently, the natural course would be to interrogate the accused himself as to the meaning of right and wrong; but this, though allowed under a commission of lunacy, would not consist with the rules of evidence in criminal cases. The question is therefore addressed to a witness, whose own estimate of right and wrong is to govern the opinion both of the judge and the jury. Let us consider how far this ought to be satisfactory. We have quoted high authority for our opinion, that there has been given to man a correct innate idea of the distinction between right and wrong, nor shall we qualify that opinion. It has been observed by a very learned modern writer, "what human actions are productive of good or of evil, what are just or unjust, or what action it is generally expedient to enforce or prohibit, are matters obviously independent of human power."* It is perfectly consistent to hold that the Divine will has allowed to us, as essential to our free agency and consequent responsibility, a just conception of the broad distinction between good and evil as abstract ideas, but has withheld from us all knowledge (except such as we may glean by observation and experience) of the process by which good or evil is worked out as the result of any given action or combination of actions. It is, indeed, often impossible, even when we witness a result, and succeed in tracing it to its causes, to say whether that result is good or the reverse, or whether the motives that set the moral machinery in motion were right or wrong. The actions of mankind cannot be reduced to these simple elements. Even when motive can be judged by the utilitarian principle alone, and where the actor in the scene is himself the arbiter, how difficult it is to determine accurately! A beggar asks me for alms, and I give him a shilling; have I acted rightly or wrongly? By giving it, I encourage mendicity and idleness; then it is wrong: by withholding it, I deprive him and his child of a necessary meal; then it is right to give: but by giving it, I provide him with the means of intemperance, and he smells of liquor already; I am clearly wrong: yet by refusing him, I may send him home penniless, though penitent, to his family, for his last farthing was expended at the gin-shop I just passed, and he now repents his folly. To refuse, would be obviously inhuman; yet by refusing, penitence may lead him to work; then it is a duty to withhold relief: but desperation may tempt him to robbery. In foro conscientiæ, I shall be accessory to his guilt, and I must give. Do I give from pure humanity?—that is right. Is my object to relieve myself from troublesome importunity?—this is extravagant and wrong. And, thus, before the catechism terminated, the beggar might die of starvation, and the catechumen be a hundred miles away.

Nor is it always easy to distinguish between mala in se and mala

* Peddie's Science of Law, 79.

quòd prohibita. A labourer's family is starving, and a hare crosses his path; he kills it, though unlicensed, and feeds his hungry children. Is the act wrong or right? The lawyer, the tax-gatherer, and the sporting magistrate are unanimous on one side, while the man himself, his fellow-labourers, and all the rate-payers, are unanimous on the other; nor will any appeal to hungry nature by the one party, or sermons on disobedience to the law by the other, disturb for a single moment the perfect conviction of both that they are right and their opponents wrong! Suppose a charge of murder, grafted on some poaching affray, and a perverted moral principle the defence; the accused is captured and disarmed, but not before he has shot the gamekeeper; he sets up this peculiar monomania, and the test is applied; he understands the guilt of murder, but he was assaulted and fired in self-defence while arrested in a rightful act—for he never could understand the guilt of poaching! The mere supposition of such a defence is preposterous to the legal ear; yet, assuming the fact to be true, and that such is the perversion of moral principle obtaining in the prisoner's mind, that he conceived himself to have been assaulted by an armed man when engaged in a legitimate pursuit of duty, is the defence unsound? If the bare suggestion of it wears a tone of ignorance and absurdity, it is because the mind is loth to receive, on mere hypothesis, a case too flimsy for discussion. In our view, such one-sided perversion of moral principle is not supposable. If we are right in our preceding remarks, the defence, to make it tenable at all, ought to go much further, and rest on the ground that the accused deemed homicide, as well as poaching, right on principle; but, as we understand the judicial dictum, this extended hallucination is not essential to relieve from responsibility, if it can be established that, as respected the provocation given, he mistook wrong for right; that being conceded as a fact, established by unquestionable evidence, he is assumed to be of diseased mind, and irresponsible for his acts. If the witnesses are fellow-labourers, and the jurymen are rate-payers, estimating his guilt by their own sense of right in such a case, the prisoner must be acquitted. A smuggler tried at Deal or Folkestone would be equally secure of impunity if defended on similar grounds. This may be bad law, but it seems necessarily to follow on the judicial definition of moral insanity.

Right and wrong, when applied to special circumstances, are arbitrary terms, and susceptible of conflicting interpretations: murder is a crime made up of circumstances; homicide may be felonious, or culpable, or justifiable, or even meritorious, according to its motive; and its motive must be deduced from circumstances. Terms so ambiguous that a judge may attach one meaning to them, a witness another, a juryman a third, and the prisoner differ from them all, can never be conveniently adopted as a test of sanity. It may be doubted if the English language could produce two words so incapable of uniform construction as "right" and "wrong;" nor would a metaphysical discussion of the nature of good and evil in open court tend much to elucidate the matter.

We would not willingly be misunderstood: if, from the evidence adduced, the judicial mind is well satisfied that a doubt exists as to the sanity of the accused, we are far from saying that the consciousness of right and wrong, in the common and familiar sense of the words, ought not to be an element taken into the account; but we mean that in all the ordinary forms of insanity known to the profession, with the exceptions already mentioned, this consciousness may consist with lunatic irresponsibility; while in that peculiar form, supposed to be occasioned by a "perversion of moral principle," the definition itself requires that the comprehension of the terms "right" and "wrong," in their ethical sense, should be sifted; but in that sense they are arbitrary, vague, and uncertain, and so involved in metaphysical obscurity, as to render them unfit for the service of a criminal court.

We have studiously avoided the common-place formality of introducing our subject by any prefatory arrangement; but, in reference to our practical object in offering this article to the public eye, it will be convenient to remind the reader that we have classified insanity by its variety of origin in the following manner:—

First, idiocy from birth; then, imbecility indicated by idiotic symptoms, not derived from native malformation, but from disorganization of the brain occasioned by disease; mania, progressing from partial delusion to total suspension of the reasoning faculty on all subjects, forms our third class; and we found a fourth in genuine monomania, arising from the habitual self-indulgence of a weak disposition. Of these four classes, we consider that the three first are invariably connected with bodily disease, while the fourth exists independently of any physical derangement, though it usually terminates in it.

A fifth class would seem to have been discovered by our criminal tribunals, under the name of "moral insanity." We have questioned, without absolutely denying the existence of such a form of mania; and, as we conceive, we have given strong, if not conclusive reasons for ranking it as extreme moral depravity, not only perfectly consistent with legal responsibility, but such as legal responsibility is expressly invented to restrain. If we are at liberty to reject this fifth class, as a spurious monomania of very rare, if not doubtful existence, we believe that it will be found that all insanity may be comprised within the other classes which we have described; and this theory, if well founded, will lead to some important practical consequences.

We have already, as we think, established one important inference—that perversion of moral principle affords no presumption of insanity, nor consciousness of right and wrong, a counter presumption of sanity; the first not being distinguishable from depravity, and the last being, at best, either an unnecessary or an uncertain test. An inquiry into the moral conduct may be useful as an auxiliary to the judgment, the total absence of shame for conduct in its nature indecent, being not unfrequently found in every class of lunacy, and sometimes a certain symptom of it: yet insensibility to shame, though utter and complete, does not of itself amount to insanity; nor does consciousness of right and wrong on ninety-nine out of a hundred subjects, prove that the reasoning faculty is unimpaired upon the hundredth. We have derived this consciousness as a gift from the Creator, simultaneously bestowed with reason; nor do we see any sufficient ground for assuming that reason may be partially affected, which does not equally sustain the argument, that consciousness of right or wrong may be partially obliterated. But does not our classification of the varieties of mania also lead to the conclusion, that, in the first place, strict inquiry into the symptoms of bodily disease is the best guide to determine sanity of mind in a doubtful case of either of the first three classes; and then, that in every class the period of time for which eccentricity has existed, and yet more, the supposed source of it, are the surest indices of its morbid character?

It is not denied that, in general, witnesses are closely interrogated on both these points, but it is seldom that due importance is given to them; on the contrary, the juryman's attention is diverted from them by elaborate, mock-scientific examinations on delusion, illusion, hallucination, and morbid imagination; and the replies are dissected with metaphysical subtlety, till the witness is as much at a loss to understand himself, as counsel or jury can be to arrive at his meaning. His evidence is mystified, and of course becomes nugatory; counsel comment upon it as suits their case; the judicial moderator is perplexed by it; the jury are bewildered, and in their confusion, think it safest to consult humanity and acquit the offender; in consequence of which, counsel, judge, and jury, and on their authority, the public, join in condemning the surgeon as a fool, properly responsible for the absurd lenity of the verdict. It is too much to expect every surgeon to be a metaphysician, or that, in walking the hospitals, we shall learn the logic of the schools. We doubt whether even the most practised logician would appear to advantage in such an academy of science as the groves of Newgate. Still, our medical brethren are not free from blame: there has been too much disposition to envelope the subject of insanity in a murky atmosphere of its own,—to assume that the mind, in its pure essence, is susceptible of disease which the body does not share, much less occasion. If this theory were just, the physician or the surgeon would be as incompetent as any stranger to the profession, to form an opinion on lunacy. We are, or ought to be, fair judges of organic or constitutional disease; it is within our province to mark the extent to which such disease affects the functions of the intellect. We may, by experience, graduate the failure of mental power by the derangement of the animal functions. Thus far we are properly within the sphere of duty. But let it be assumed that the mind is capable of disorder apart from all bodily ailment, what do we know of it more than others? the mind has no pulsation—no dyspnœa, no dyspepsia—no outward and visible signs from which we can deduce its disorder. It is true that the physician has more abundant opportunity of observing the shapes and forms that mental aberration may assume; but though he may truly say that he has known this or the other visionary idea to be prevalent in some class of mania, he will not say that he has in any case felt competent to decide on the existence of mania by the prevalence of the visionary idea alone; he determines the patient to be maniacal from other symptoms: the visionary idea must be associated with irregularity of the animal functions, to satisfy his mind. Restlessness is perhaps the only indication of mental disorder which partakes of bodily disease, when no derangement of the animal system can be detected by the usual signs; yet restlessness is no certain criterion, even if it were possible for a medical witness to judge of its source, unless he had long been in daily attendance. A man may be restless from anxiety, from habits of impatience, or from indulgence in sanguine expectations; and yet there may be no mania. We are not yet provided with any unequivocal test of mental malady by which we can judge of the failure of reason, unless in cases where the mind is sympathetically affected with the body. As a general proposition, disease of mind alone has no symptoms apart from the body; if in its pure and abstracted essence, it admits of disorder, which metaphysically may be a question of much doubt, medical men have no better means of predicating the nature or extent of the derangement than the unlearned, and it is more honest to avow it.

We so far blame our profession, that they affect to know more than the principles of the science can possibly convey; but at the same time, we feel persuaded that, by a judicious classification of mental derangement, as it is usually indicated by morbid bodily symptoms, they may qualify themselves to give a sound and useful opinion in our courts of law, even in cases where they are called upon to speak, not from personal observation of the supposed maniac, but from the report of others. We have considered it our duty to call attention to what seems to be the fallacy of the judicial test of mania, but it is more with the view of inducing the medical world to consider the subject as its importance deserves, that we have suggested a principle for the classification of mental disease by its origin, duration, and especially by its attendant physical symptoms. On such matters we ought to be at home: on others, "ne sutor ultra crepidam."

We may, perhaps, be charged with forgetting the maxim ourselves, if, in conclusion, we repeat a suggestion that has been before made, for the improvement of our criminal practice in cases involving the issue of lunacy. A sufficient precedent is not wanting for the trial of such an issue by a separate jury specially qualified. Where a woman pleads pregnancy as a ground for deferring capital punishment, a jury of matrons is impanelled to try that plea. A recent case has certainly thrown much doubt on the competency of such a tribunal; but it cannot be denied that on this very difficult question they are less likely to err than a jury of the other sex. Why should not a similar course be pursued where lunatic irresponsibility is claimed on a prisoner's behalf? The issue here is involved in far more obscurity, and the decision of it is acknowledged to rest principally on medical judgment; the varieties of mental alienation are so numerous, that though we may succeed on the principles which we have explained, in tracing them up to one of the four classes that have been mentioned, and so far in simplifying the subject, it is hopeless to attempt throwing light upon it from the witness-box in the hurried confusion of a criminal trial, involving, perhaps, twenty different questions of law and fact. In the very effort which we have here made to divest the subject of the technicalities of science, and render it intelligible to ordinary readers, the difficulty of compressing our remarks within two hours' reading will be apparent; yet we have passed over without observation all the peculiarities of delirium, delusion, derangement, eccentricity, partial development, hysterical affection, intermittent mania, suspended volition, fatuity, incipient dotage, casual aberration, suspended memory, and many other generic terms by which the profession are wont to describe mania in all its phases. Is it, then, reasonable to expect that time shall be allowed in open court to discuss as well as to explain all the shades and gradations, by which the physician draws his diagnosis between the analogous symptoms of every class? or that even if time were not restricted, he could render his explanations in perspicuous language to men who, however learned and sagacious on other topics, are generally unskilled in the terms of art, and wholly inexperienced in the delicacy of observation by which alone such nice distinctions can be satisfactorily drawn?

If, in addition to this, the medical witness is required to trace each variety to its source, to enter into the causes and character of all cerebral disease or malformation, to explain the pathology of nervous disorders, or scrofulous habit, or other constitutional taint or intemperate propensity, he necessarily preaches in an unknown tongue without the gift of interpretation; and to add difficulty to his dilemma, he preaches to an audience whose duty it is to be sceptical, and to question, if not oppose, his dogmas with all the subtlety of forensic power.

Surely, in such cases, common sense dictates the expediency of a change such as we have proposed; but if the precedent is followed, it should be with this alteration—the issue of lunacy should be the first tried, not merely because if found in favour of the accused he ought not, needlessly, to be subjected to a second trial, but because his guilt or his innocence, so far as other circumstances must determine it, not being within the cognizance of the same jury, they will consider their verdict with a consciousness of relief from all the painful consequences that may possibly follow; if their judgment establishes sanity, they incur no ultimate responsibility beyond putting the prisoner on his trial. Such an arrangement would also consist with an important relaxation of the law of evidence to which we have before adverted; a man may not be questioned if his answers may criminate himself. On a trial for lunacy, however, it is mercy to a man to afford him the opportunity. If really insane, the more closely he is examined, the more likely is his malady to appear. If his answers establish his insanity, he does not convict himself of guilt, but proves his innocence; while, of course, it would be provided for his protection, that his statement or even confession should not be admissible evidence against him, if a verdict of sane mind should be returned. Nor would it be the least of the advantages to be gained by the proposed change of system, that the public mind would cease to be agitated by doubts on the propriety of a verdict in equivocal cases of sanity.

The present commissioners of lunacy would naturally appear to be the proper judges on such a mesne inquiry, and, aided by a special jury of medical men, would command public confidence; but we forbear. It is enough to throw out the suggestion, in the hope that it will be taken up by those who are fully competent, by influence as well as talent, to regenerate our criminal jurisprudence.

Since this article was in type, the report of the important case of Clift *v.* Schwabe, argued in the Exchequer Chamber, has reached us. It is too late to be made the subject of observation, but it is a case calculated to confirm our views of the superficial manner in which insanity is generally discussed in our courts of law.

